# Integrated Bioinformatics and Validation Reveal Potential Biomarkers Associated With Progression of Primary Sjögren’s Syndrome

**DOI:** 10.3389/fimmu.2021.697157

**Published:** 2021-07-23

**Authors:** Ning Li, Lei Li, Mengyao Wu, Yusi Li, Jie Yang, Yicheng Wu, Haimin Xu, Danyang Luo, Yiming Gao, Xiaochun Fei, Liting Jiang

**Affiliations:** ^1^ Department of Stomatology, Ruijin Hospital, Shanghai Jiao Tong University School of Medicine, College of Stomatology, Shanghai Jiao Tong University, Shanghai, China; ^2^ Department of Pathology, Ruijin Hospital, Shanghai Jiao Tong University School of Medicine, Shanghai, China; ^3^ Core Facility of Basic Medical Sciences, Shanghai Jiao Tong University School of Medicine, Shanghai, China

**Keywords:** primary Sjogren’s syndrome (pSS), transcriptome sequencing, potential biomarkers, bioinformatics analysis, severity

## Abstract

**Background:**

Primary Sjögren’s syndrome (pSS) is a chronic systemic autoimmune disease of the exocrine glands characterized by specific pathological features. Previous studies have pointed out that salivary glands from pSS patients express a unique profile of cytokines, adhesion molecules, and chemokines compared to those from healthy controls. However, there is limited evidence supporting the utility of individual markers for different stages of pSS. This study aimed to explore potential biomarkers associated with pSS disease progression and analyze the associations between key genes and immune cells.

**Methods:**

We combined our own RNA sequencing data with pSS datasets from the NCBI Gene Expression Omnibus (GEO) database to identify differentially expressed genes (DEGs) *via* bioinformatics analysis. Salivary gland biopsies were collected from 14 pSS patients, 6 non-pSS patients, and 6 controls. Histochemical staining and transmission electron micrographs (TEM) were performed to macroscopically and microscopically characterize morphological features of labial salivary glands in different disease stages. Then, we performed quantitative PCR to validate hub genes. Finally, we analyzed correlations between selected hub genes and immune cells using the CIBERSORT algorithm.

**Results:**

We identified twenty-eight DEGs that were upregulated in pSS patients compared to healthy controls. These were mainly involved in immune-related pathways and infection-related pathways. According to the morphological features of minor salivary glands, severe interlobular and periductal lymphocytic infiltrates, acinar atrophy and collagen in the interstitium, nuclear shrinkage, and microscopic organelle swelling were observed with pSS disease progression. Hub genes based on above twenty-eight DEGs, including MS4A1, CD19, TCL1A, CCL19, CXCL9, CD3G, and CD3D, were selected as potential biomarkers and verified by RT-PCR. Expression of these genes was correlated with T follicular helper cells, memory B cells and M1 macrophages.

**Conclusion:**

Using transcriptome sequencing and bioinformatics analysis combined with our clinical data, we identified seven key genes that have potential value for evaluating pSS severity.

## Introduction

Primary Sjögren’s syndrome (pSS) is a chronic systemic autoimmune disease of the primary exocrine glands characterized by specific pathological features (1, 2). pSS often features immune-mediated destruction of exocrine tissues, such as salivary and lacrimal glands, resulting in the loss of saliva and tear production (3). Immunological abnormalities such as antinuclear antibodies (ANAs), antibodies against Ro/Sjögren’s syndrome-related antigen A (SSA) and La/Sjögren’s syndrome-related antigen B (SSB), and hypergammaglobulinemia are often detected in pSS patients by laboratory tests (4, 5). pSS is estimated to affect between 0.03 and 4.8% of individuals across different regions and global populations and occurs in women approximately 9 to 20 times more frequently than in men (6–8). However, the pathogenesis and underlying mechanism of pSS are still unclear due to the heterogeneity of clinical phenotypes and complex pathogenetic mechanisms. Therefore, identifying disease-associated biomarkers is necessary to understand the complex progression of pSS.

The hallmark characteristic histological feature of pSS is the infiltration T cells, B cells, plasma cells, macrophages, mast cells, and dendritic cells into the lacrimal and salivary glands (9). Salivary glands from pSS patients express a unique profile of cytokines, adhesion molecules, and chemokines compared to healthy controls (10). Nocturne et al. revealed that T lymphocytes are key factors in the immunopathogenesis of pSS and that the activation of B cells accelerates disease progression and drives some disease-specific phenotypes (11). Current studies have increased the understanding of autoimmune disease complexity. Although the innate immune system has been demonstrated to play a significant role in early stages of pSS, the exact role played by regulatory cells and their related genes in SS is far from understood. Labial minor salivary gland (LSG) biopsy is widely used in the diagnosis of pSS due to its simplicity, familiarity, and acceptance, and plays an irreplaceable role in the established American-European Consensus Group classification criteria and the proposed American College of Rheumatology/European League Against Rheumatism (ACR/EULAR) criteria (12). Evidence suggests that investigation of LSGs could reveal potential biomarkers and additional prognostic, stratification, or mechanistic insights in pSS (13).

Previous Literature has reported that flow cytometry or immunohistochemistry can be used to detect certain immune cell subsets and genes related to pSS in peripheral blood mononuclear cells (PBMCs) and salivary glands (14). Over the past decade, genetic studies have been a useful and effective tool for the identification of novel pathogenesis-supporting pathways. Nocturne et al. reviewed the first genome-wide association study (GWAS) and identified three major pathogenic steps in pSS: innate immune system activation, B-cell activation, and T-cell activation (11). Using RNA sequencing data and four salivary glands microarray datasets, Luo et al. revealed the potential roles of ICOS in pSS (15). Recently, single-cell RNA sequencing has been applied to PBMCs from pSS patients, to identify the immune cell subsets and susceptibility genes related to the pathogenesis of pSS (16). However, little is known about genes associated with disease progression. Overall, delineating the molecular events and related genes involved in different stages of pSS represents an unmet clinical need, as this information could improve disease diagnosis and inform future therapeutic target selection.

Herein, we performed a comprehensive investigation involving transcriptomic analyses, salivary histology, and validation of hub genes in salivary gland samples from different stages of pSS. First, guided by integrated bioinformatics analysis, we identified differentially expressed genes (DEGs) from both our own RNAseq data (minor salivary gland) and pSS datasets (both major and minor salivary glands) from the NCBI Gene Expression Omnibus (GEO) database. Second, histochemical staining and transmission electron micrographs (TEM) were performed to macroscopically and microscopically characterize the morphological features of minor salivary glands at different disease stages. Furthermore, we performed quantitative PCR to validate hub genes. Finally, we analyzed the correlations between selected hub genes and immune cells in the autoimmune microenvironment. Our findings provide further insight into the mechanisms underlying the progression of pSS and suggest that the hub genes are potential diagnostic biomarkers and therapeutic targets.

## Materials and Methods

### Data Download and Preprocessing

The “GEOquery” package in R software was used to download the pSS datasets GSE40611 (17), GSE127952 (https://www.ncbi.nlm.nih.gov/geo/query/acc.cgi?acc=GSE127952), and GSE154926 (https://www.ncbi.nlm.nih.gov/geo/query/acc.cgi?acc=GSE154926), and GSE159574 (15) was downloaded from the corresponding supplementary file from the Gene Expression Omnibus (GEO, http://www.ncbi.nlm.nih.gov/geo/). The microarray datasets, GSE40611 and GSE127952, contained 17 pSS and 10 control parotid tissue samples and eight pSS and six control minor salivary gland samples, respectively. For these datasets, the normalize between arrays function in the R limma package was used to remove the inter-batch difference effects, and the maximum value method was used to remove the duplicate gene names corresponding to multiple probes. The high-throughput sequencing count datasets, GSE154926 and GSE159574, contained 43 pSS and 7 control minor salivary gland samples, and 16 pSS and 13 non-pSS salivary gland samples, respectively. For these datasets, the R Bioconductor package DESeq2 was used for data standardization and to obtain the standardized matrix file.

### Bioinformatics Analysis

The DEGs from GSE40611, GSE127952, and GSE154926 were identified using the limma and DEseq2 packages (|logFC| >1, p<0.05) and using as training cohorts. The “ClueGO” plug-in with integration of Gene Ontology (GO) terms in Cytoscape was used to construct separate biological process networks from the list of upregulated genes (18). Gene Ontology (GO) annotation and Kyoto Encyclopedia of Genes and Genomes (KEGG) pathway analyses were performed using the cluster profiler package in R (19). P-value <0.05 was set as the cutoff criterion for significance. Interactive relationships and protein–protein interaction (PPI) networks of the overlapping DEGs were evaluated using the STRING database (http://string-db.org) (20). Cytoscape software (http://www.cytoscape.org/) was used to construct and visualize a biological network of key DEGs (21). A receiver operating characteristic (ROC) curve was used to analyze diagnostic values, including the area under the curve (AUC), sensitivity, and specificity obtained from the GSE159574 dataset. CIBERSORT (http://cibersort.stanford.edu) (22) was used to calculate immune cell proportion and estimate the scores of 22 human immune cell types in pSS samples from the GSE154926 cohort (p<0.05). Spearman correlation analysis was performed to assess correlations between hub genes and immune cells.

### RNA Sequencing Data

We selected three cases each of pSS and non-pSS and sequenced each group of labial minor salivary gland tissue pools separately. Total RNA of each group was extracted by TRIzol Reagent (Invitrogen, USA) and quantified and qualified by Agilent 2100 Bioanalyzer (Agilent Technologies, CA, USA), NanoDrop (Thermo Fisher Scientific Inc.), and 1% agarose gel. One microgram total RNA with RIN > 6.5 was used for following library preparation. Next-generation sequencing library preparations were constructed according to the manufacturer’s protocol. Then the prepared libraries were subsequently multiplexed and loaded on an Illumina HiSeq instrument (Illumina, CA, USA). Sequencing was carried out using a 2 × 150 bp paired-end (PE) configuration. Image analysis and base calling were conducted by the HiSeq Control Software (HCS), OLB, and GAPipeline-1.6 (Illumina) on the HiSeq instrument. To remove technical sequences, pass filter data of fastq format were processed by Cutadpt (V1.9.1) to be high-quality clean data. Differential expression analysis used the DESeq2 Bioconductor package. Padj of genes were set at <0.05 to detect differential expressed ones. The raw data were uploaded to the NCBI SRA database, under accession the number PRJNA722690.

### Clinical Samples

Labial salivary glands from a total of 14 pSS patients with pSS, 6 non-pSS subjects (female, 40–65 years old), and 6 healthy control patients (mucous cysts or lower lip trauma) were collected from Ruijin Hospital, Shanghai Jiao Tong University School of Medicine (Shanghai, China). All pSS patients fulfilled either the 2016 ACR/EULAR classification criteria (23) or the 2012 ACR classification criteria (24) for pSS. Non-pSS meet the same diagnostic criteria as patients presenting with xerostomia and xerophthalmia, but do not meet the classification criteria for pSS (25). The labial salivary gland biopsies were divided into four groups based on lymphocyte infiltration levels (25): healthy control, non-pSS, pSS1 (lymphatic infiltration foci = 1), and pSS2 (lymphatic infiltrating foci ≥ 2). Tissue samples for histochemical staining were fixed immediately following resection and were embedded in paraffin after 24 h. The samples for transmission electron micrographs (TEM) were fixed with 2.5% glutaraldehyde in a 0.1 M phosphate‐buffered saline (PBS) for 2 h at 4°C. Other biopsy samples were flash frozen in liquid nitrogen and stored at −80°C until RNA extraction.

### Histochemical Staining

Hematoxylin and eosin (HE) staining, Alcian blue (AB)-Sirius red staining, and Alcian blue-Periodic Acid-Schiff (PAS) stained sections from labial salivary glands of both non-SS and pSS subjects were examined under a light microscope (Leica, DMLB, Leica Microsystems Wetzlar, Germany). Inflammatory infiltration (grading and scoring), fibrosis (diffuse or partial), acinar atrophy with ductal epithelial changes, and amyloid deposits were assessed by independent observers. Tissue damage arising from both ducts and acini, lymphocyte infiltrates, fibrosis, etc., were also analyzed (26, 27).

### Transmission Electron Micrographs (TEM)

All non-SS and pSS labial salivary glands samples for transmission electron microscopy were subjected to 2.5% glutaraldehyde fixation, 1% osmium tetroxide postfixation, and ethanol gradient dehydration. Two changes of 100% propylene oxido (P.O.) for 10 minutes each and finally into the embedding resin. The samples were sectioned by diamond knife (LEICA EM UC7) to 70-90nm, followed by electron stained with lead citrate, and visualized on a Transmission electron microscope (HITACHI H-7650).

### RNA Extraction and Quantitative RT-PCR

RNA was extracted using Takara RR420 and RR036A isolation kits according to the manufacturer’s instructions. First-strand cDNA synthesis was performed using PrimeScript reverse transcriptase (Takara RR420). qPCR was carried out using the Takara RR036A kit. β-Actin was used as an internal reference. The relative expression of target genes was calculated using 2^−ΔΔCt^ method. A P value <0.05 was considered significant. Primer sequences are summarized in **Table S1**. All PCR reactions were conducted in triplicate.

### Immunohistochemical Staining of Biomarkers in Labial Salivary Glands

Based on the RT-PCR results, we selected two established proteins representing T cells and B cells, for validation. After deparaffinization, paraffin-embedded non-pSS, pSS1, and pSS2 labial salivary gland sections were incubated in antigen retrieval solution (Dako, Denmark), followed by the appropriate blocking steps. Following CD3 (IR503, Dako, Denmark) and CD19 (IR656, Dako, Denmark) primary antibody staining, slides were incubated with HRP-conjugated secondary antibody and developed with DAB (Dako, Denmark). Fourteen pSS labial salivary glands and 6 non-pSS tissues were chosen randomly. Images were captured under a light microscope (Olympus Bx51, Japan).

### Statistical Analysis

Statistical analysis was performed using unpaired Student’s t-test or one-way ANOVA in GraphPad Prism software. Data are presented as means ± SD. A P value < 0.05 was considered statistically significant.

## Results

### Identification of Common DEGs in pSS

**Figure 1:**
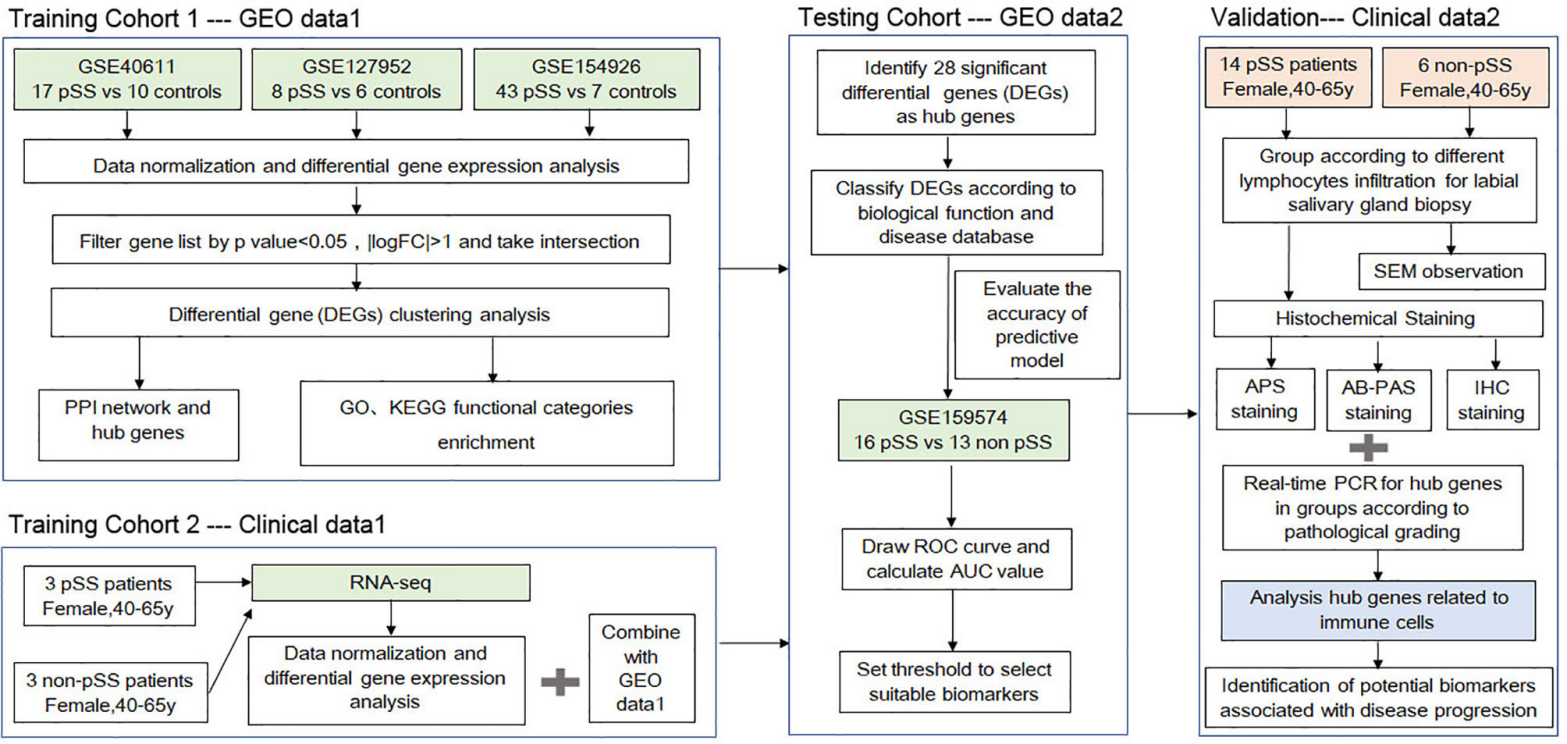
A schematic showing the protocol utilized in the current study to identify the potential biomarkers associated with the progression of primary Sjögren’s syndrome (pSS).

**Figure 2:**
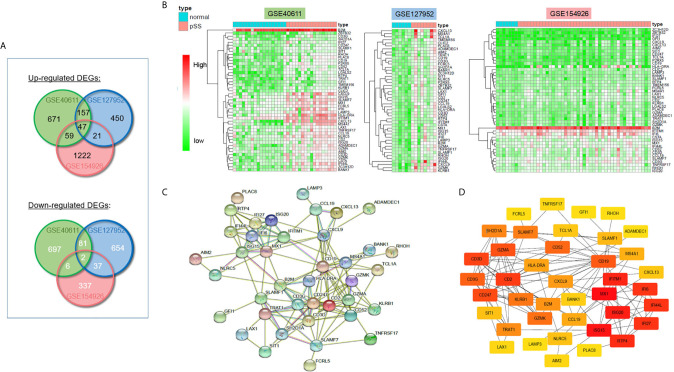
Differentially expressed genes (DEGs) in pSS and normal samples from the GSE40611, GSE127952, and GSE154926 datasets. **(A)** Venn diagrams showing the overlap between significant DEGs from the control and pSS samples within each dataset. **(B)** Heatmaps of common DEG (≥2 datasets overlap) pSS *vs.* control samples in three GSE datasets. **(C)** Construction of the PPI network based on common DEGs (≥3 datasets overlap). **(D)** Construction of DEG networks using the DEGREE algorithm in the Cytoscape software.

The workflow of this study is shown in **Figure 1**. To investigate the common DEGs involved in pSS, we downloaded three public pSS datasets, including major (parotid) salivary gland (GSE40611) and minor salivary gland (GSE127952, GSE154926) datasets. As seen in **Figure 2A** and **Table S2**, a total of 47 upregulated and 2 downregulated common DEGs (≥3 instances of intersection) were identified (|logFC| >1, p<0.05). **Figure 2B** indicates whether these DEGs were found in the non-pSS *versus* pSS samples from each of three datasets. To systematically analyze the relationships between the common DEGs, we constructed a PPI network using the STRING database and visualized the data using Cytoscape 3.4.0 (**Figure 2C**). In addition, a DEG network was constructed using DEGREE algorithm analysis within the Cytoscape software (**Figure 2D**). Here, the hub nodes, including CD3G, CD3D, CD2, B2M, CD19, MS4A1, CXCL9, CXCL13, IFI6, GZMK, et al., were considered hub genes in the pSS DEG list. The identification of these DEGs laid the foundation for the subsequent identification of potential pSS biomarkers.

### Function and Pathway Enrichment Analysis of Common Upregulated DEGs

**Figure 3:**
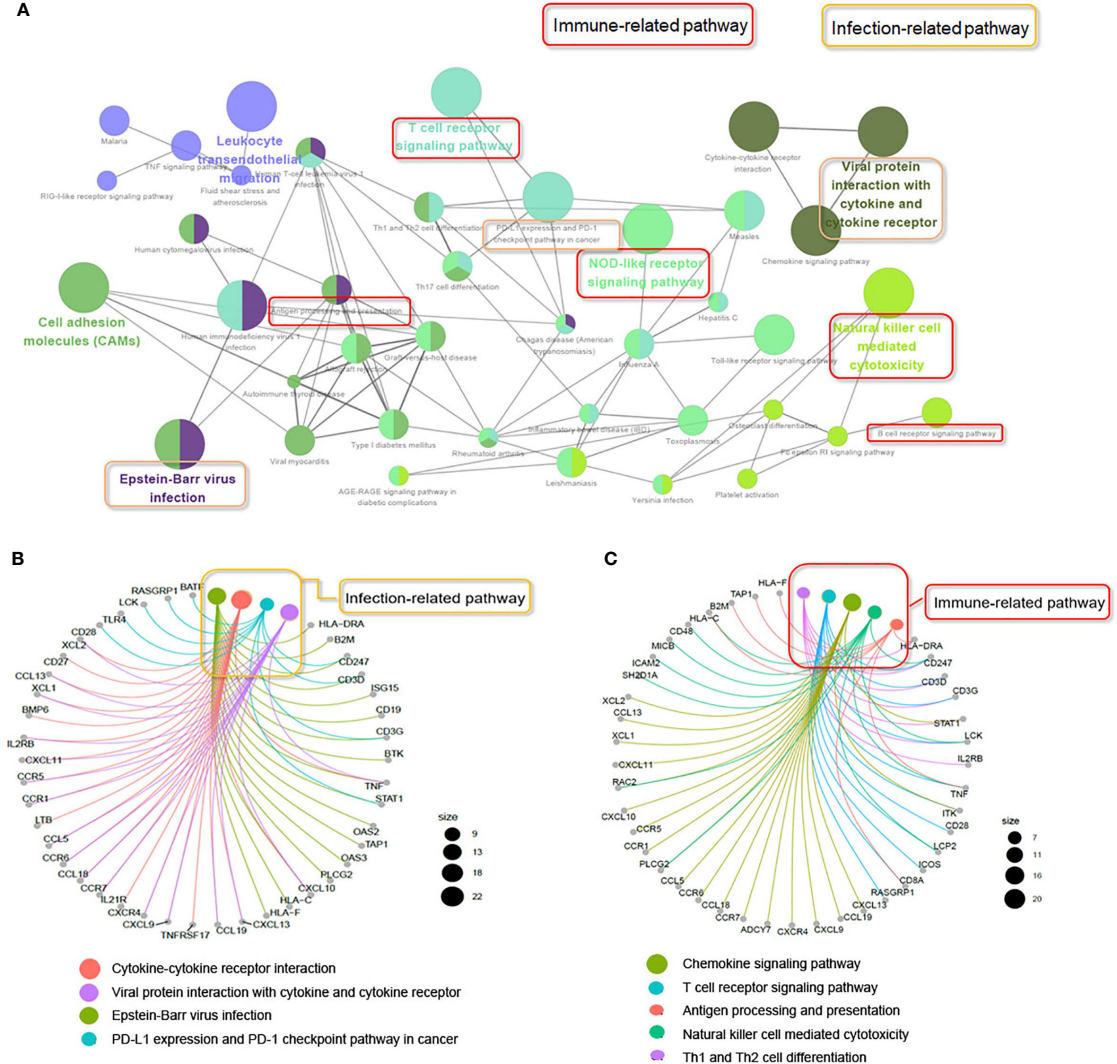
ClueGO network analysis of the upregulated common DEGs. **(A)** KEGG pathway analyzes the upregulated common DEGs (≥2 times of intersection genes) involved in the occurrence and development of pSS. The pathways in the red box indicate the immune-related signaling pathways. The pathway in the yellow box indicates the infection-related signaling pathways. **(B)** Genes involved in infection-related signaling pathways. **(C)** Genes involved in immune-related signaling pathways.

**Figure 4:**
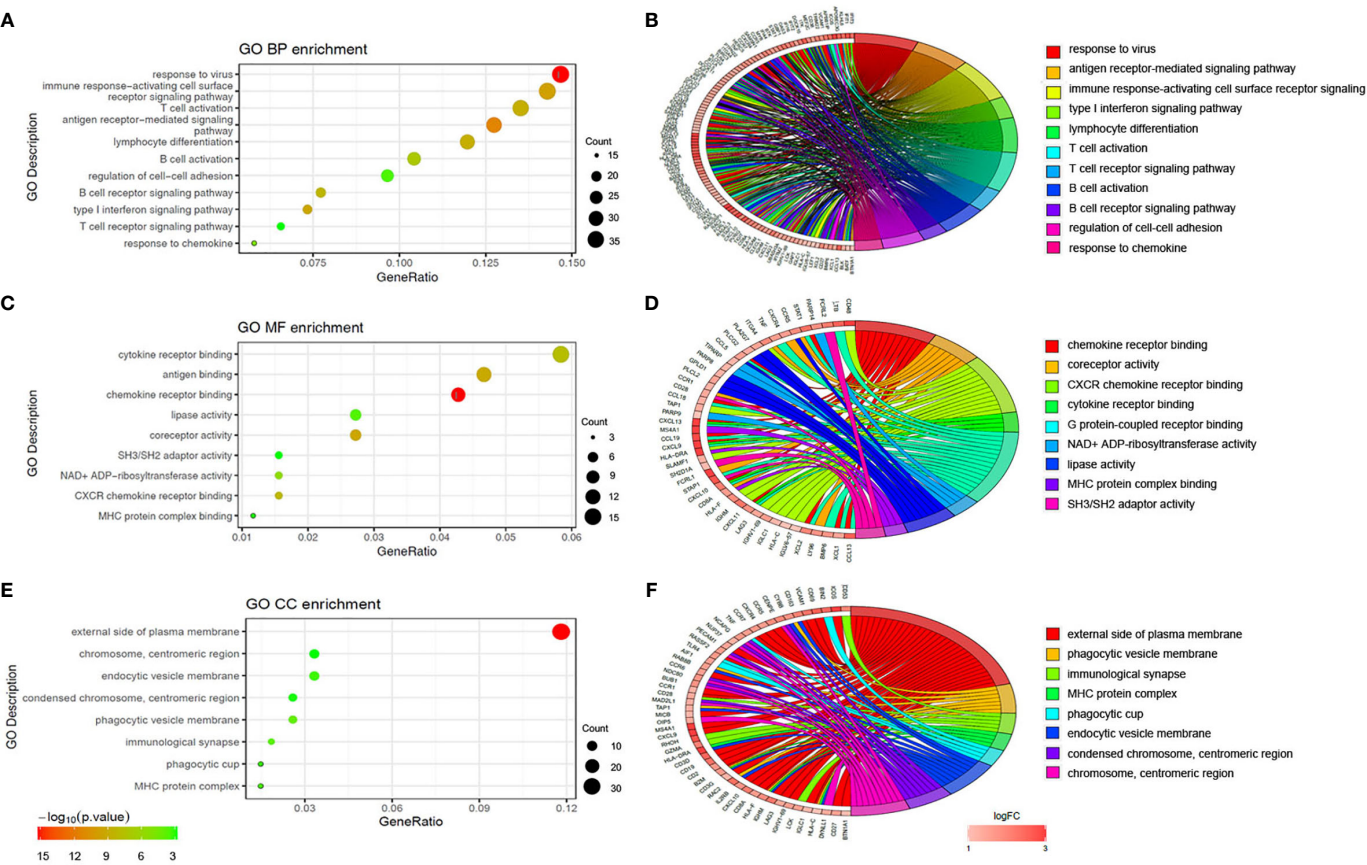
Functional enrichment analysis of upregulated DEGs. The significantly enriched GO-biological processes **(A–B)**, GO-cellular component **(C–D)**, and GO-molecular function **(E–F)** of the upregulated DEGs.

To explore the potential biological processes associated with pSS, we performed pathway analysis on the common upregulated DEGs using the ClueGO plug-in within the Cytoscape suite (18). As seen in **Figure 3A**, we observed enrichment of immune-related signaling pathways like chemokine signaling pathway, T cell receptor signaling pathway, antigen processing and presentation, natural killer cell–mediated cytotoxicity, and Th1 and Th2 cell differentiation. Viruses that lead to inflammation, deregulation of epithelial cells, and autoimmune responses have been implicated as one of the pathogenic drivers of pSS (9). In line with previous studies, our findings demonstrated that common DEGs were also involved in infection-related pathways such as Epstein-Barr virus infection and viral protein interactions with cytokines and cytokine receptors. The pathway-related genes are displayed in **Figures 3B, C**. Using the DAVID database, the top five GO terms related biological processes among DEGs were primarily associated with response to virus, immune response-activating cell surface, receptor signaling pathway, T cell activation, antigen receptor-mediated signal pathway, and lymphocyte differentiation (**Figures 4A, B** and **Table S3**). The terms related to molecular functions were mainly involved in cytokine receptor binding, antigen binding, and chemokine receptor binding (**Figures 4C, D** and **Table S4**). In addition, there is significant correlation in external side of plasma membrane, chromosome, centromeric region, and endocytic vesicle membrane in relation to cellular component (**Figures 4E–F** and **Table S5**). These genes could be related to multiple biological pathways orchestrating pSS pathogenesis.

### Identifying Candidate Biomarkers of pSS

**Figure 5:**
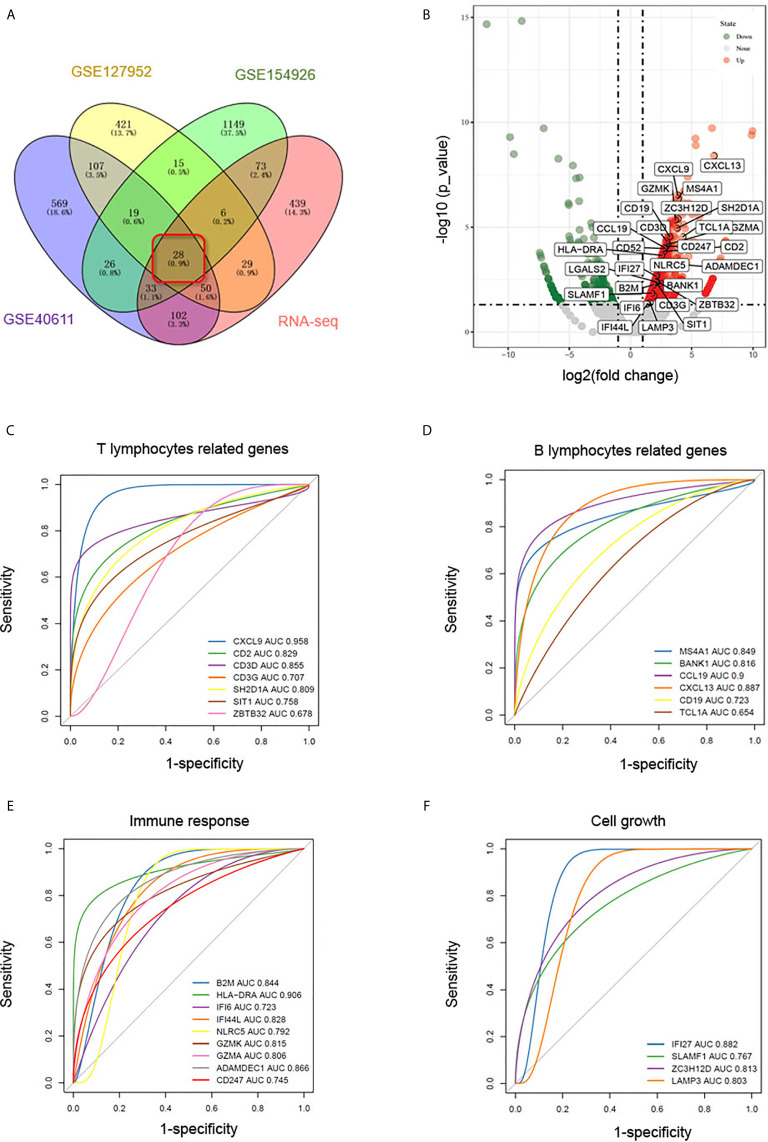
Identification of biomarkers in common DEGs. **(A)** Venn diagram showing common DEGs (logFC > 1, p < 0.05) to the GSE127952, GSE154926, and GSE40611 datasets and our RNA-Seq data. **(B)** Volcano map of the 28 common DEGs in our RNA-seq data. In the volcano plots, green and red points represent downregulated and upregulated genes, respectively, with logFC > 1, p < 0.05. **(C–F)** ROC curves based on pSS dataset GSE159574 predict candidate biomarkers related to T lymphocytes **(C)**, B lymphocytes **(D)**, immune response **(E)**, and cell growth **(F)**.

RNA-Seq was carried out to further validate the results of the bioinformatics analyses above. As shown in **Figures 5A, B**, 28 DEGs were identified as common to the GSE127952, GSE154926, GSE40611, and our RNA-Seq data and therefore may be useful as candidate pSS biomarkers. To further verify the accuracy of these selected DEGs, another pSS dataset (GSE159574) was used as testing cohort to generate ROC curve. As shown in **Figures 5C–E**, common DEGs associated with T lymphocytes (CXCL9, CD3D, CD3G, CD2, SH2D1A, SIT1, ZBTB32), B lymphocytes (MS4A1, BANK1, CD19, TCL1A, CXCL13, CCL19), immune response (B2M, HLA-DRA, IFI6, IFI44L, NLRC5, GZMK, GZMA, ADAMDEC1, and CD247), and cell growth (IFI27, SLAMF1, ZC3H12D, and LAMP3) were further assessed (AUC > 0.65). These common DEGs were thought to be potential biomarkers of pSS.

### Histological Observation of pSS Progression

**Figure 6:**
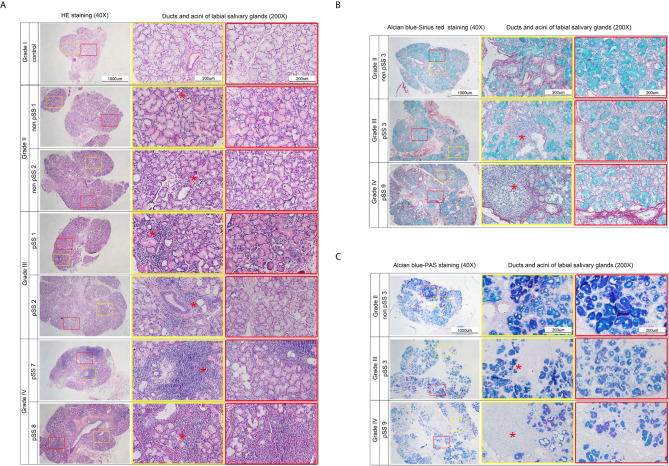
Representative histochemical staining microphotographs in the labial salivary glands revealing pSS-specific histopathology of different stages. **(A)** Sections represent slight infiltrate (Grade I), moderate infiltrate or less than one focus (Grade II), one focus, mainly located in periductal region (Grade III), and more than one focus with a heavy interlobular and periductal lymphocytic infiltrate (Grade IV). (Hematoxylin and eosin stain, original magnification ×40 or ×200.) **(B)** Sections represent that in control group the acinar (blue) is abundant, the structure is clear, and the collagen (red) in the acinar interstitium is evenly distributed (Grade II). With the severity of pSS lesions, the serous and mucous acini appeared atrophied, and the blue and blue-purple stained areas are significantly reduced. Severe acinar atrophy and collagen in the interstitium increases, and fat infiltration appears in Grade IV. (Alcian blue-Sirius red stain, original magnification × 40 or × 200.) **(C)** Sections represent that under normal circumstances, acidic mucous substances are blue, glycogen and neutral mucous substances are red, and mixed mucous substances are blue-purple or purple-blue. As the degree of SS disease increases, serous and mucous acini appear atrophied. The blue and blue-violet stained areas are significantly reduced (Alcian blue-PAS stain, original magnification×40 or ×200). *Red asterisk indicates the lymphocyte infiltration. Focus indicates Lymphocytic infiltrate of >50 cells. Sections represent slight infiltrate (Grade I), moderate infiltrate or less than one focus (Grade II), one focus, mainly located in periductal region (Grade III), and more than one focus with a heavy interlobular and periductal lymphocytic infiltrate (Grade IV). (Hematoxylin and eosin stain, original magnification ×40 or ×200).

As a limited amount of information is available regarding the link between macroscopic and microscopic features of the disease progression, we performed morphological observations to explore changes to the acini and ducts during various disease stages. Clinical characteristics of the pSS patients are shown in **Table 1**. The labial salivary gland samples were divided according to their lymphocyte infiltration levels for labial salivary gland biopsy (25). Macroscopically, as seen in **Figure 6A**, we could clearly observe slight infiltration (Grade I); moderate infiltration or less than one focus (Grade II); one focus, mainly located in the periductal region (Grade III); and more than one focus with heavy interlobular and periductal lymphocytic infiltration (Grade IV). In addition, Alcian blue-Sirius red staining revealed abundant acinar tissue (blue) with clear cellular structures as well as evenly distributed collagen (red) in the acinar interstitium (Grade II) of non-pSS samples. pSS lesions of increasing severity, the serous and mucous acini appeared atrophied, and the blue and blue-purple stained areas were significantly reduced. In Grade IV samples, we noted severe acinar atrophy, increased interstitial collagen, and the appearance of fat infiltration **(** **Figure 6B** **)**. Alcian blue-PAS staining showed that under normal circumstances, acidic mucous substances were blue, a glycogen and neutral mucous substances were red, and mixed mucous substances were blue-purple or purple-blue. As pSS disease severity increased, serous and mucous acini appeared atrophied, and blue and blue-violet stained areas were significantly reduced (**Figure 6C**). Representative histochemical staining of the labial salivary glands revealed pSS stage–specific histopathology stages and suggested that related genes may also change significantly in severe pSS patients.

**Table 1:** Clinical characteristics of pSS female patients included in this study.

ID number	Age (years)	ANA	Anti-SSA	Anti-SSB	Anti-Ro-52 antibody	Dry mouth	Dry eyes
**Grade III**	**27**	**1:80+**	**+**	**-**	**++**	**+**	**+**
	**40**	**1:320+**	**++**	**-**	**-**	**+**	**+**
	**31**	**1:80+**	**-**	**+**	**-**	**+**	**+**
	**69**	**1:160+**	**-**	**-**	**-**	**+**	**+**
	**56**	**1:160+**	**-**	**-**	**++**	**+**	**+**
	**40**	**1:80+**	**-**	**-**	**±**	**+**	**+**
**Grade IV**	**54**	**1:320+**	**+**	**-**	**+**	**+**	**+**
	**50**	**1:1,280+**	**++**	**±**	**++**	**+**	**+**
	**62**	**1:160+**	**++**	**-**	**++**	**+**	**+**
	**58**	**1:80+**	**-**	**-**	**++**	**+**	**+**
	**55**	**1:160+**	**++**	**±**	**++**	**+**	**+**
	**25**	**1:320+**	**++**	**-**	**-**	**+**	**+**
	**54**	**1:160+**	**++**	**-**	**++**	**+**	**+**
	**35**	**1:80+**	**+**	**+**	**++**	**+**	**+**

### Scanning Electron Microscopy (SEM)

**Figure 7:**
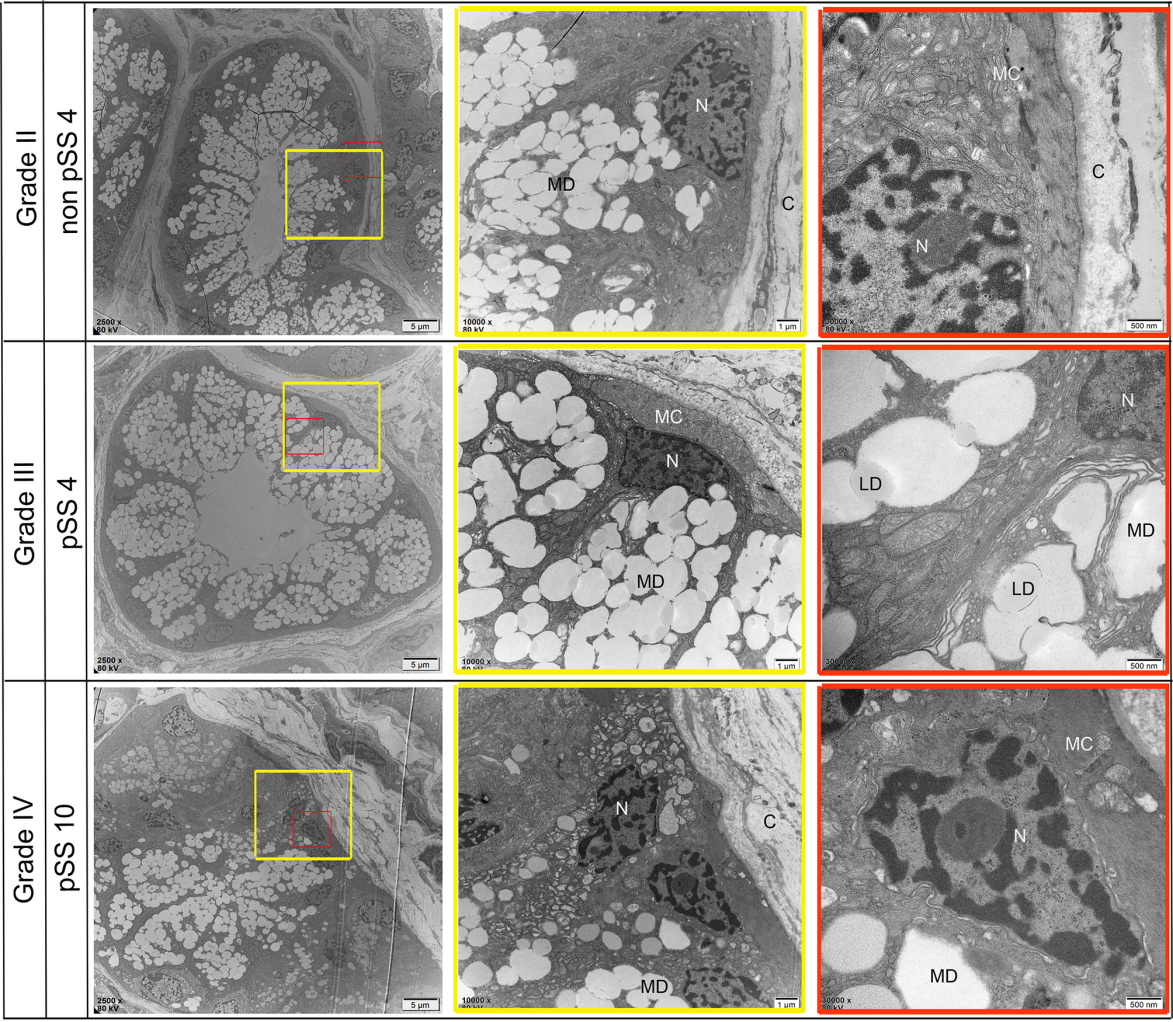
Representative transmission electron micrograph (TEM) images of acinar-specific ultrastructure changes in the labial salivary gland of non-pSS and pSS. In non-pSS (Grade II), the structure of the acinar is normal, and the glandular epithelial cells in the acinar are myoepithelial cells. The cytoplasmic skeletal components and their unique myofilaments could be seen. With the aggravation of pSS, there is serous and mucous acini atrophy, glandular epithelial cell nucleus shrinkage, organelle swelling to varying degrees, and increase of interstitial collagen components. MD, Mucous Droplets; N, Nucleus; MC, myoepithelial cell; C, collagen; LD, lipid droplets. Bar: 5 μm, 1 μm, 500 nm.

**Figure 8:**
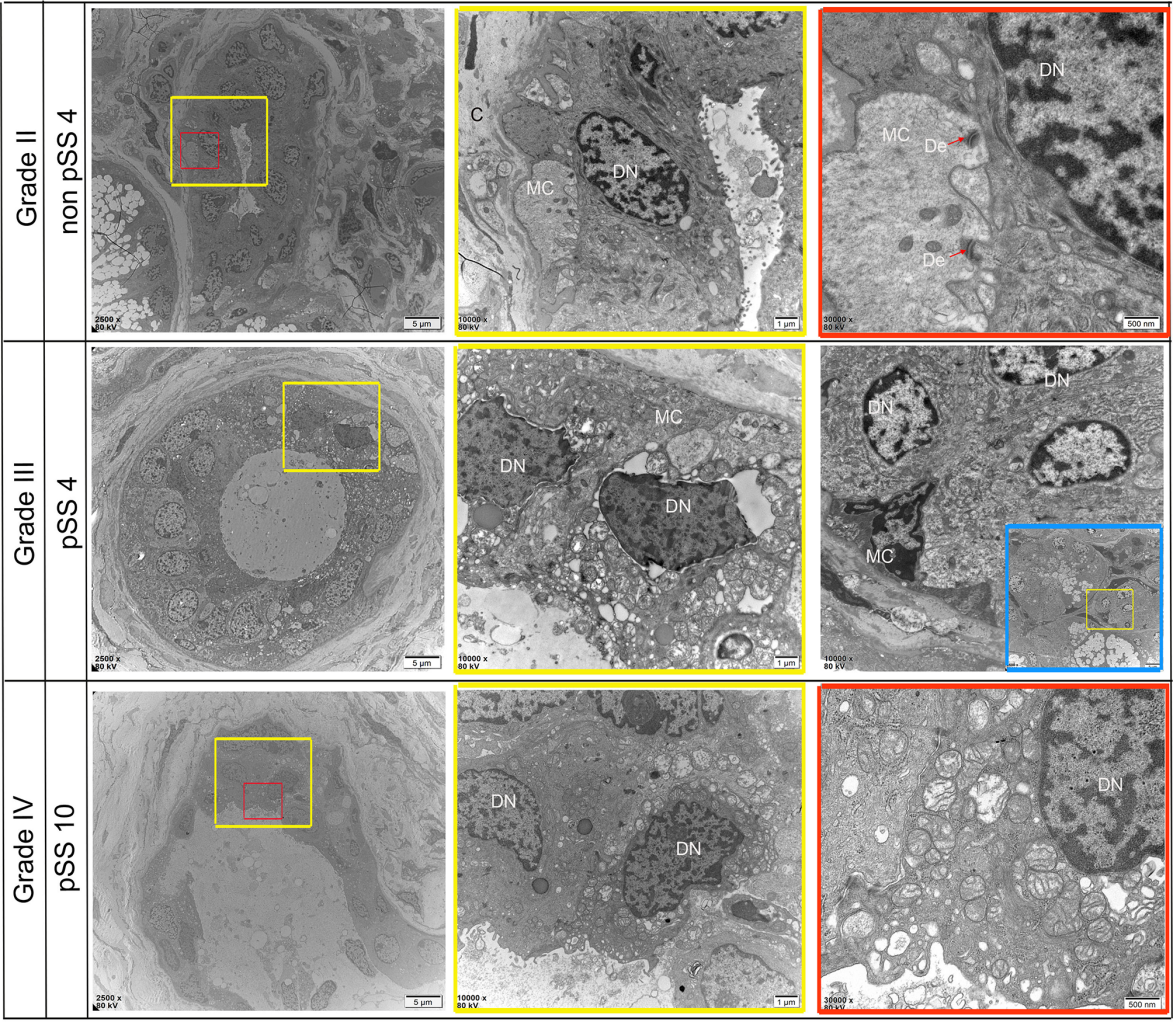
Representative transmission electron micrograph (TEM) images of dust-specific ultrastructure changes in the labial salivary gland of non-pSS and pSS. In non-pSS (Grade II), the duct structure of normal glands is clear, and ductal epithelial cells have full nuclei and are connected with myoepithelial cells by desmosomes. As the degree of SS disease increases, duct structures begin to atrophy and become disordered, and part of the basement membrane damage disappears. Ductal epithelial cells can see obvious nuclear pyknosis, fat infiltration in the cytoplasm, and swelling of intracytoplasmic organelles. MC, myoepithelial cell; DN, Ductal Epithelial Cells Nucleus; De, desmosome. Bar, 5 μm, 1 μm, 500 nm

Ultrastructural alterations were detected in the labial salivary glands of pSS and non-pSS. In non-pSS (Grade II) sections, the acinar cell structure was normal, and the glandular epithelial cells in the acinus were myoepithelial cells. Cytoskeletal components and their unique myofilaments could be seen. By SEM we observed nuclear pyknosis of the duct epithelial cells and obvious swelling of organelles in pSS samples (Grade III) compared with non-pSS samples (Grade II) (**Figure 7**). In severe (grade IV) pSS, we observed disordered ductal structures, disappearance of some basal cells, obvious nuclear pyknosis of the duct epithelial cells, fat infiltration into the cytoplasm, and swelling of intracytoplasmic organelles (**Figure 8**). To our knowledge, this is the first study to report the microscopic labial gland changes during pSS disease progression.

### Identifying Potential Biomarkers Associated With pSS Procession

**Figure 9:**
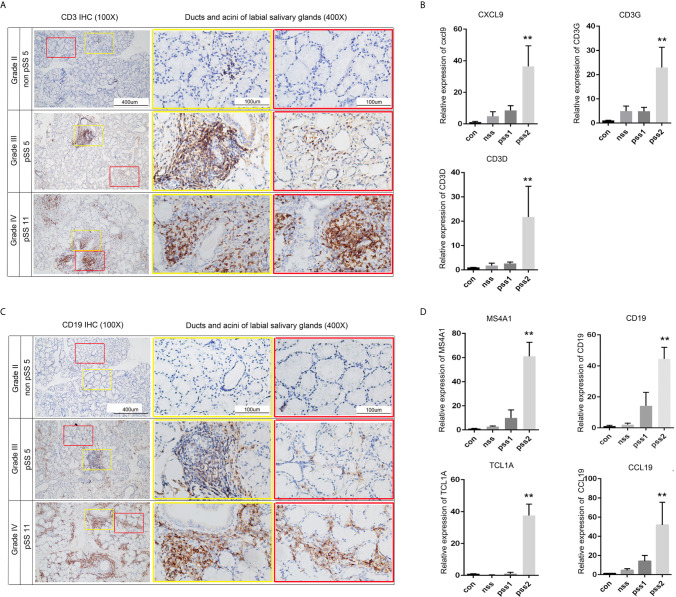
Validation of potential biomarkers associated with pSS progression. **(A)** CD3 staining (brown) of sequential sections show slight infiltration of T cells (Grade II) or large T cell aggregates with periductal infiltration (Grade III and Grade IV) (original magnification ×100 or ×400). **(B)** Statistical analysis of the real-time PCR results for T-cell-related genes: CXCL9, CD3G, and CD3D. Gene expression in pSS2 (Grade IV) samples is shown relative to control, non-pSS (Grade II), and pSS1 (Grade III) samples. **(C)** CD19 staining (brown) of sequential sections slight (Grade II), moderate (Grade III) infiltration of B cells, and large periductal and interlobular infiltration (Grade IV) (original magnification × 100 or × 400). **(D)** Statistical analysis of the real-time PCR results for B-cell-related genes: MS4A1, CD19, TCL1A, and CCL19. Gene expression in pSS2 (Grade IV) samples is shown relative to control, non-pSS (Grade II), and pSS1 (Grade III). All data are presented as mean ± SD of results. **p < 0.01, as calculated by Student’s t-test.

Although recent advances in high-throughput sequencing have provided the opportunity to identify disease-related genes in blood or tissue samples, relatively little is known about genes associated with disease progression. To explore potential biomarkers associated with pSS stages and disease progression, we collected labial minor salivary gland biopsy samples from pSS patients and divided them into four groups according to lymphocyte infiltration. Subsequently, we performed RT-PCR to validate the expression levels of the abovementioned 28 DEGs. Of these, we selected seven hub genes (**Figures 9B, D**) that were significantly higher in pSS2 samples (p<0.01) than in control, non-pSS, and pSS1 samples, including MS4A1, CD19, TCL1A, CCL19, CXCL9, CD3G, and CD3D (over 10-fold increase) (data for other genes not shown). In accordance with the results of previous reports and our own RT-PCR, we observed increased T cell (CD3^+^) and B cell (CD19^+^) infiltration in pSS2 (Grade IV) samples (**Figures 9A, C**).

### Correlation Between Potential Biomarkers and Immune Cells

**Figure 10:**
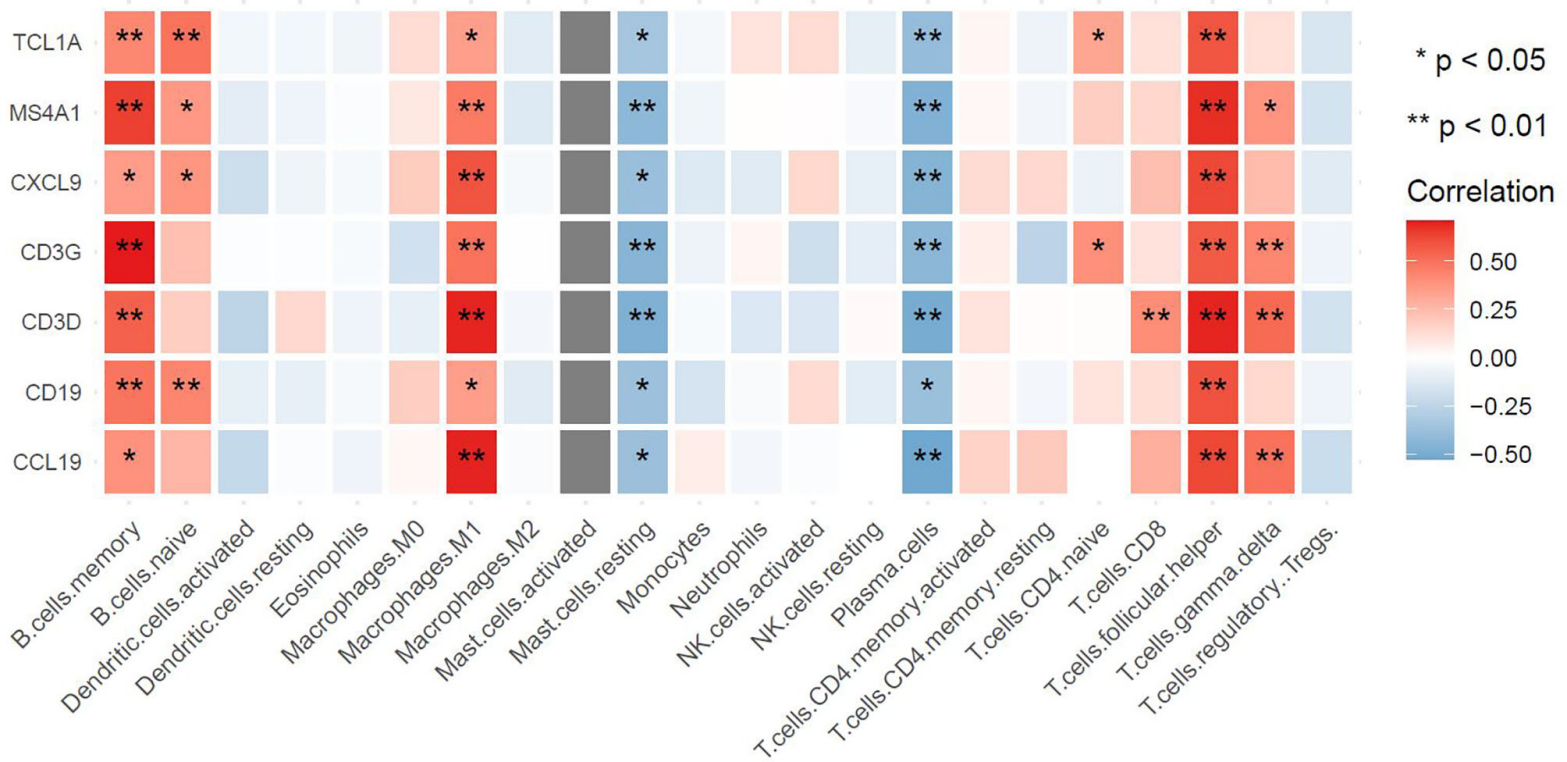
Associations between hub genes and immune cells as defined by the CIBERSORT algorithm. The horizontal axis represents immune cell contents, and the vertical axis represents genes. Different colors represent the correlation coefficient (red represents positive correlation, blue represents negative correlation), with a darker color indicating a stronger correlation. *p < 0.05, **p < 0.01, the asterisk represents the degree of importance.

The above results demonstrated that the hub genes were highly enriched in immune-related pathways and infection-related pathways. To explore the underlying mechanisms associated with these potential biomarkers, we extracted the gene expression matrix from the GSE154926 pSS samples and used the CIBERSORT algorithm to estimate correlations between these genes and infiltration of pSS samples with 22 human immune cell types. As shown in **Figure 10**, the potential biomarkers MS4A1, CD19, TCL1A, CCL19, CXCL9, CD3G, and CD3D were positively correlated with T follicular helper cells (Tfh cells), memory B cells, and M1 macrophage infiltration. This result indicated a strong correlation between the seven potential biomarkers and the immune response.

## Discussion

PSS is one of the more prevalent autoimmune diseases. The clinical presentation of pSS can differ greatly, ranging from common sicca symptoms to systemic symptoms (such as arthralgias, fatigue, neuropathy, or vasculitis) (28). PSS is a disease characterized by lymphocytic-mediated exocrine gland inflammation (29–31); however, the underlying genetic mechanisms driving the pathogenesis of pSS remain unclear. Lymphocytic infiltration of the exocrine glands, which leads to impaired lacrimal and salivary secretion, is the main characteristic of pSS (32). Although diagnostic criteria such as disordered saliva secretion have been widely used for pSS classification, their sensitivity still demands further improvement (33). The lack of standardized criteria used for diagnosis and classification of the disease leads to difficulty interpreting epidemiologic studies. In addition, a previous study reported that pSS patients typically do not receive a final diagnosis until as much as 7 years after the onset of symptoms (34). Therefore, the identification of potential biomarkers for pSS diagnosis is critical.

Previous studies have extensively investigated molecular biomarkers of pSS over the last several decades; however, these studies were conducted based on only one kind of gland (3, 8, 35–37). Considering the complexity of underlying pSS occurrence and development, it is essential for us to consider the expression of genes from all salivary glands in combination with clinical verification to identify more convincing biomarkers. In this study, we combined public datasets from major and minor salivary gland samples to screen for common DEGs as potential biomarkers. A total of 47 upregulated common DEGs were identified as likely to be involved in pSS development. The impact of DEGs is often to initiate fundamental molecular changes. To better appreciate the underlying mechanism of pSS, functional enrichment analysis was performed. The upregulated common DEGs were enriched in KEGG terms associated with immune-related pathways and infection-related pathways, including the T cell receptor and B cell receptor signaling pathways. GO terms implicated in pSS pathogenesis include immune system process (38), T cell activation (39–41), B cell activation (42, 43), and response to virus (44, 45). Previously reported pSS-related genes, including HLA-DRA (8, 46), CCL19 (47, 48), CXCL9 (49), and CD3D (50), were enriched in our study, suggesting that the related common DEGs are potential biomarkers for pSS. RNA-Seq was subsequently carried out to explore diagnostic biomarkers. The 28 common DEGs were thought to be directly involved in pSS development.

Investigating progression-associated gene expression profiles might enhance our understanding of the mechanisms underlying pSS progression. The public datasets we utilized for our bioinformatics analyses included transcriptomic profiles of both the major and minor salivary glands. To further screen for novel potential biomarkers associated with pSS progression and diagnosis, we collected labial salivary gland biopsy samples from 14 pSS and 6 non-pSS patients. We then investigated acinar and ductal morphologies at both the macroscopic and microscopic levels at various disease stages. Macroscopically, the occurrence of foci (an aggregate >50 lymphocytes), lymphocytic infiltration of the duct walls, and several other abnormalities (such as scattered lymphocyte infiltrates, interstitial sclerosis, and dilatation and regression of ducts and acini) are important features for the classification of pSS (51, 51). Infiltration of lymphocytes into the salivary gland, which leads to destruction and subsequent fibrotic changes, is typically observed in pSS patients (52, 53). Compared with non-pSS samples (Grade I, II), pSS samples clearly displayed one (Grade III) or more (Grade IV) foci. Microscopically, the development of pSS correlated with severe interlobular and periductal lymphocytic infiltration, increased acinar atrophy, and interstitial collagen and nuclear shrinkage and organelle swelling. To our knowledge, this is the first report using TEM to investigate the microscopic changes in pSS labial glands and the correlation of these changes with disease progression.

T lymphocytes are considered to be key factors in the immunopathogenesis of pSS, and the activation of B cells accelerates the disease process and produces some disease-specific manifestations (11). In accordance with the enriched KEGG signaling pathways identified here, immunohisto- chemistry revealed that B cells and T cells are significantly increased around the ducts and acini of pSS samples compared with non-pSS samples. T lymphocytes (16, 54) and B lymphocytes (55, 56) were predominantly found around the ducts and within the lymphocytic infiltrates of pSS biopsy samples. Activated T cells contribute to disease pathogenesis by producing proinflammatory cytokines, which leads to the establishment of a positive feedback loop (57). Our observations demonstrate that pSS progression is associated with T/B cells, which is in accordance with functional enrichment analysis results (GO:0042110, GO:0050852, GO:0042113, and GO:0050853). Therefore, we selected common DEGs related to T cells (CXCL9, CD3D, CD3G) and B cells (MS4A1, CD19, TCL1A, CCL19) as potential biomarkers and verified these by qRT-PCR. Furthermore, we estimated positive correlations between potential biomarkers and Tfh cells, memory B cells, and M1 macrophages using the CIBERSORT algorithm. We suggest that heightened immune activation might broadly influence inflammatory infiltrates, which might subsequently influence the expression of DEGs.

MS4A1 (58) and CD19 (59) are B-lymphocyte-specific membrane protein-coding genes related to immunodeficiency. These genes play significant roles in the regulation of cellular calcium influx, development, differentiation, and activation of B lymphocytes. Mudd et al. indicated that MS4A1 is expressed in subsets of T cells and exhibits significantly higher expression in nivolumab-bound T cells from human patients undergoing anti-PD-1 immunotherapy (60), which is in accordance with the results of our functional enrichment analysis. MS4A1 has also been identified in a study published by Luo et al. (15). CD19+ B cells are often used for the identification of pSS and have been widely verified as a diagnostic marker (61, 62). CCL19 maybe involved in more sever forms of autoimmune diseases, such as rheumatoid arthritis (63). Carubbi et al. indicated that CCL19 is involved in pSS pathogenesis (64), and Liu et al. identified CCL19 as a potential biomarker of pSS disease progression (47). CXCL9 has been shown to take part in T-cell trafficking, and Aota et al. found that enhanced production of CXCL9 may contribute to anti-inflammatory functions in pSS lesions (49). TCL1A is associated with precursor T-cell acute lymphoblastic leukemia and prolymphocytic leukemia (65). CD3G/CD3D forms the T-cell receptor-CD3 complex. Defects in CD3G are associated with T cell immunodeficiency (66), while CD3D is involved in T-cell development and signal transduction (67). Moreover, TCL1A is a novel biomarker that has never been reported in pSS. MS4A1, CD19, TCL1A, CCL19, CXCL9, CD3G, and CD3D might be potential diagnostic biomarkers associated with the progression of pSS, which remains to be verified based on a larger sample.

## Conclusion

Our study describes characteristic pathological and ultrastructural changes in the salivary glands of severe pSS patients, including serous and mucous acinar atrophy, shrinkage of glandular epithelial cell nuclei, and damage to ductal structures and the basement membrane. Furthermore, using transcriptomic sequencing and bioinformatics analysis combined with our clinical data, we identified seven hub genes (MS4A1, CD19, TCL1A, CCL19, CXCL9, CD3G, and CD3D) that have potential value for the evaluation of pSS severity and that were strongly correlated with the immune response in the autoimmune microenvironment. Overall, understanding the mechanisms of pSS disease progression may pave the way for novel therapeutic interventions.

## Data Availability Statement

The original contributions presented in the study are publicly available. This data can be found here: https://www.ncbi.nlm.nih.gov/bioproject/PRJNA722690/.

## Ethics Statement

The studies involving human participants were reviewed and approved by Ethics Committee of Ruijin Hospital, School of Medicine, Shanghai Jiaotong University. The patients/participants provided their written informed consent to participate in this study.

## Author Contributions

LJ and YG designed the overall research strategy. NL, LL, MW, YL, JY, YW, HX, DL, and XF performed the experiments. NL and LJ analyzed the data and wrote the manuscript. All authors contributed to the article and approved the submitted version.

## Funding

This work was supported by National Natural Science Foundation of China (NSFC No. 81900975).

## Conflict of Interest

The authors declare that the research was conducted in the absence of any commercial or financial relationships that could be construed as a potential conflict of interest.

## Publisher’s Note

All claims expressed in this article are solely those of the authors and do not necessarily represent those of their affiliated organizations, or those of the publisher, the editors and the reviewers. Any product that may be evaluated in this article, or claim that may be made by its manufacturer, is not guaranteed or endorsed by the publisher.

## References

[1] Seror R Ravaud P Bowman S Baron G Tzioufas A Theander E. EULAR Sjogren’s Syndrome Disease Activity Index: Development of a Consensus Systemic Disease Activity Index for Primary Sjogren’s Syndrome. Ann Rheumatic Dis (2010) 69(6):1103–9. doi: 10.1136/ard.2009.110619 PMC2937022 19561361

[2] Manuel R Pilar B Raphaèle S Hendrika B Simon JB Thomas D. Characterization of Systemic Disease in Primary Sjögren’s Syndrome: EULAR-SS Task Force Recommendations for Articular, Cutaneous, Pulmonary and Renal Involvements. Rheumatol (Oxf Engl) (2017) 56(7):1245. doi: 10.1093/rheumatology/kex157 PMC7191868 28379527

[3] Oyelakin A Horeth E Song E Min S Che M Marzullo B. Transcriptomic and Network Analysis of Minor Salivary Glands of Patients With Primary Sjögren’s Syndrome. Front Immunol (2020) 11:606268. doi: 10.3389/fimmu.2020.606268 33488608 PMC7821166

[4] Rivière E Pascaud J Virone A Dupré A Ly B Paoletti A. Interleukin-7/Interferon Axis Drives T Cell and Salivary Gland Epithelial Cell Interactions in Sjögren’s Syndrome. Arthritis Rheumatol (Hoboken NJ) (2020) 73(4):631–40. doi: 10.1002/art.41558 33058491

[5] Bodewes I Björk A Versnel M Wahren-Herlenius M. Innate Immunity and Interferons in the Pathogenesis of Sjögren’s Syndrome. Rheumatol (Oxford Engl) (2019) 60(6):2561–73. doi: 10.1093/rheumatology/key360 30770713

[6] Xavier M Francois H. Renal Involvement in Primary Sjogren Syndrome. Nat Rev Nephrol (2016) 12(2):82–93. doi: 10.1038/nrneph.2015.174 26568188

[7] Gabriel M Cynthia S Eric L Divi C. Prevalence of Primary Sjgren’s Syndrome in a US Population-Based Cohort. Arthritis Care Res (2017) 69(10):1612–6. doi: 10.1002/acr.23173 PMC5478481 27998024

[8] Zhang L Xu P Wang X Zhang Z Zhao W Li Z. Identification of Differentially Expressed Genes in Primary Sjögren’s Syndrome. J Cell Biochem (2019) 120(10):17368–77. doi: 10.1002/jcb.29001 31125139

[9] Psianou K Panagoulias I Papanastasiou AD de Lastic AL Rodi M Spantidea PI. Clinical and Immunological Parameters of Sjogren’s Syndrome. Autoimmun Rev (2018) 17(10):1053–64. doi: 10.1016/j.autrev.2018.05.005 30103041

[10] Hansen A Reiter K Ziprian T Jacobi A Hoffmann A Gosemann M. Dysregulation of Chemokine Receptor Expression and Function by B Cells of Patients With Primary Sjogren’s Syndrome. Arthritis Rheum (2005) 52(7):2109–19. doi: 10.1002/art.21129 15986367

[11] Nocturne G Mariette X. Advances in Understanding the Pathogenesis of Primary Sjögren’s Syndrome. Nat Rev Rheumatol (2013) 9(9):544–56. doi: 10.1038/nrrheum.2013.110 23857130

[12] Fox RI. Standardisation of Labial Salivary Gland Biopsies in Sjogren’s Syndrome: Importance for the Practicing Rheumatologist. Ann Rheumatic Dis (2017) 76(7):1159–60. doi: 10.1136/annrheumdis-2016-210851 28254788

[13] Fisher BA Brown RM Bowman SJ Barone F. A Review of Salivary Gland Histopathology in Primary Sjogren’s Syndrome With a Focus on its Potential as a Clinical Trials Biomarker. Ann Rheumatic Dis (2015) 74(9):1645–50. doi: 10.1136/annrheumdis-2015-207499 26034044

[14] Luo J Ming B Zhang C Deng X Li P Wei Z. IL-2 Inhibition of Th17 Generation Rather Than Induction of Treg Cells Is Impaired in Primary Sjogren’s Syndrome Patients. Front Immunol (2018) 9:1755. doi: 10.3389/fimmu.2018.01755 30150979 PMC6100298

[15] Luo J Liao X Zhang L Xu X Ying S Yu M. ICOSTranscriptome Sequencing Reveals Potential Roles of in Primary Sjögren’s Syndrome. Front Cell Dev Biol (2020) 8:592490. doi: 10.3389/fcell.2020.592490 33344450 PMC7747463

[16] Hong X Meng S Tang D Wang T Ding L Yu H. Single-Cell RNA Sequencing Reveals the Expansion of Cytotoxic CD4 T Lymphocytes and a Landscape of Immune Cells in Primary Sjögren’s Syndrome. Front Immunol (2020) 11:594658. doi: 10.3389/fimmu.2020.594658 33603736 PMC7884617

[17] Horvath S Nazmul-Hossain ANM Pollard RPE Kroese FGM Vissink A Kallenberg CGM. Systems Analysis of Primary Sjögren’s Syndrome Pathogenesis in Salivary Glands Identifies Shared Pathways in Human and a Mouse Model. Arthritis Res Ther (2012) 14(6):R238. doi: 10.1186/ar4081 23116360 PMC3674589

[18] Gabriela B Bernhard M Hubert H Pornpimol C Marie T Amos K. ClueGO: A Cytoscape Plug-In to Decipher Functionally Grouped Gene Ontology and Pathway Annotation Networks. Bioinformatics (2009) 25(8):1091–3. doi: 10.1093/bioinformatics/btp101 PMC2666812 19237447

[19] Yu G Wang LG Han Y He QY. Clusterprofiler: An R Package for Comparing Biological Themes Among Gene Clusters. Omics: J Integr Biol (2012) 16(5):284–7. doi: 10.1089/omi.2011.0118 PMC3339379 22455463

[20] Damian S Andrea F Stefan W Kristoffer F Davide H Jaime HC. STRING V10: Protein–Protein Interaction Networks, Integrated Over the Tree of Life. Nucleic Acids Res (2015) 43(D1):447–52. doi: 10.1093/nar/gku1003 PMC4383874 25352553

[21] Kohl M Wiese S Warscheid B. Cytoscape: Software for Visualization and Analysis of Biological Networks. Methods Mol Biol (2011) 696:291–303. doi: 10.1007/978-1-60761-987-1_18 21063955

[22] Newman A Liu C Green M Gentles A Feng W Xu Y. Robust Enumeration of Cell Subsets From Tissue Expression Profiles. Nat Methods (2015) 12(5):453–7. doi: 10.1038/nmeth.3337 PMC4739640 25822800

[23] Vitali. Classification Criteria for Sjogren’s Syndrome: A Revised Version of the European Criteria Proposed by the American-European Consensus Group. Ann Rheum Dis (2002) 61(6):554–8. doi: 10.1136/ard.61.6.554 PMC1754137 12006334

[24] Shiboski S Shiboski C Criswell L Baer A Challacombe S Lanfranchi H. American College of Rheumatology Classification Criteria for Sjögren’s Syndrome: A Data-Driven, Expert Consensus Approach in the Sjögren’s International Collaborative Clinical Alliance Cohort. Arthritis Care Res (2012) 64(4):475–87. doi: 10.1002/acr.21591 PMC3349440 22563590

[25] Chisholm DM Mason DK. Labial Salivary Gland Biopsy in Sjögren’s Disease. J Clin Pathol (1968) 21(5):656–60. doi: 10.1136/jcp.21.5.656 PMC473887 5697370

[26] Skarstein K Aqrawi L Øijordsbakken G Jonsson R Jensen J. Adipose Tissue Is Prominent in Salivary Glands of Sjogren’s Syndrome Patients and Appears to Influence the Microenvironment in These Organs. Autoimmunity (2016) 49(1/8):338–46. doi: 10.1080/08916934.2016.1183656 27206986

[27] Aqrawi LA Jensen JL Oijordsbakken G Ruus AK Nygard SL Holden M. Signalling Pathways Identified in Salivary Glands From Primary Sjgren’s Syndrome Patients Reveal Enhanced Adipose Tissue Development. Autoimmunity (2018) 51(3):135–46. doi: 10.1080/08916934.2018.1446525 29504848

[28] Patel R Shahane A. The Epidemiology of Sjogren’s Syndrome. Clin Epidemiol (2014) 6:247–55. doi: 10.2147/CLEP.S47399 PMC4122257 25114590

[29] Mariette X Criswell LA. Primary Sjögren’s Syndrome. N Engl J Med (2018) 378(10):931–9. doi: 10.1056/NEJMcp1702514 29514034

[30] Masaki Y Sugai S. Lymphoproliferative Disorders in Sjögren’s Syndrome. Autoimmun Rev (2004) 3(3):175–82. doi: 10.1016/S1568-9972(03)00102-2 15110228

[31] Ramos-Casals M Brito-Zerón P Sisó-Almirall A Bosch X. Primary Sjogren Syndrome. BMJ (2012) 344:e3821. doi: 10.1136/bmj.e3821 22700787

[32] Price HP Hodgkinson MR Curwen RS Maclean LM Brannigan JA Carrington M. The Orthologue of Sjögren’s Syndrome Nuclear Autoantigen 1 (SSNA1) in Trypanosoma Brucei Is an Immunogenic Self-Assembling Molecule. PloS One (2012) 7(2):e31842. doi: 10.1371/journal.pone.0031842 22363749 PMC3282761

[33] Liang P Zhu W Lan T Tao Q. Detection of Salivary Protein Biomarkers of Saliva Secretion Disorder in a Primary Sjögren Syndrome Murine Model. J Pharm BioMed Anal (2018) 154:252–62. doi: 10.1016/j.jpba.2018.03.023 29558726

[34] Segal B Bowman SJ Fox PC Vivino FB Mclean L. Primary Sjgren’s Syndrome: Health Experiences and Predictors of Health Quality Among Patients in the United States. Health Qual Life Outcomes (2009) 7(1):46. doi: 10.1186/1477-7525-7-46 19473510 PMC2693523

[35] Shi H Cao N Pu Y Xie L Zheng L Yu C. Long non-Coding RNA Expression Profile in Minor Salivary Gland of Primary Sjögren’s Syndrome. Arthritis Res Ther (2016) 18(1):109. doi: 10.1186/s13075-016-1005-2 27188286 PMC4869341

[36] Aqrawi LA Galtung HK Vestad B Øvstebø R Thiede B Rusthen S. Identification of Potential Saliva and Tear Biomarkers in Primary Sjögren’s Syndrome, Utilising the Extraction of Extracellular Vesicles and Proteomics Analysis. Arthritis Res Ther (2017) 19(1):14. doi: 10.1186/s13075-017-1228-x 28122643 PMC5264463

[37] Inamo J Suzuki K Takeshita M Kassai Y Takiguchi M Kurisu R. Identification of Novel Genes Associated With Dysregulation of B Cells in Patients With Primary Sjögren’s Syndrome. Arthritis Res Ther (2020) 22(1):153. doi: 10.1186/s13075-020-02248-2 32571405 PMC7310138

[38] Martin-Gutierrez L Peng J Thompson N Robinson G Naja M Peckham H. Two Shared Immune Cell Signatures Stratify Patients With Sjögren’s Syndrome and Systemic Lupus Erythematosus With Potential Therapeutic Implications. Arthritis Rheumatol (Hoboken NJ) (2021). doi: 10.1002/art.41708 33645922

[39] Pontarini E Verstappen G Grigoriadou S Kroese F Bootsma H Bombardieri M. Blocking T Cell Co-Stimulation in Primary Sjögren’s Syndrome: Rationale, Clinical Efficacy and Modulation of Peripheral and Salivary Gland Biomarkers. Clin Exp Rheumatol (2020) 4):222–7. 33095146

[40] Wang Y Chen S Chen J Xie X Gao S Zhang C. Germline Genetic Patterns Underlying Familial Rheumatoid Arthritis, Systemic Lupus Erythematosus and Primary Sjögren’s Syndrome Highlight T Cell-Initiated Autoimmunity. Ann Rheumatic Dis (2020) 79(2):268–75. doi: 10.1136/annrheumdis-2019-215533 31848144

[41] Stergiou I Papageorgiou A Chatzis L Tzioufas A Voulgarelis M Goules A. T Cell Lymphoma in the Setting of Sjögren’s Syndrome: T Cells Gone Bad? Report of Five Cases From a Single Centre Cohort. Clin Exp Rheumatol (2020) 4):125–9. 33025901

[42] Rivière E Pascaud J Tchitchek N Boudaoud S Paoletti A Ly B. Salivary Gland Epithelial Cells From Patients With Sjögren’s Syndrome Induce B-Lymphocyte Survival and Activation. Ann Rheumatic Dis (2020) 79(11):1468–77. doi: 10.1136/annrheumdis-2019-216588 32843324

[43] Sun J Zhang H Liu S Lian C Chen Z Shao T. Elevated EPSTI1 Promote B Cell Hyperactivation Through NF-κb Signalling in Patients With Primary Sjögren’s Syndrome. Ann Rheumatic Dis (2020) 79(4):518–24. doi: 10.1136/annrheumdis-2019-216428 32114510

[44] Chang C Yen Y Lee C Lin C Huang C Tsai C. Lower Risk of Primary Sjogren’s Syndrome in Patients With Dengue Virus Infection: A Nationwide Cohort Study in Taiwan. Clin Rheumatol (2021) 40(2):537–46. doi: 10.1007/s10067-020-05282-2 PMC7817565 32671658

[45] van Nimwegen J van der Tuuk K Liefers S Verstappen G Visser A Wijnsma R. Vaginal Dryness in Primary Sjögren’s Syndrome: A Histopathological Case-Control Study. Rheumatol (Oxf Engl) (2020) 59(10):2806–15. doi: 10.1093/rheumatology/keaa017 PMC7516088 32044981

[46] Fang K Zhang K Wang J. Network-Assisted Analysis of Primary Sjögren’s Syndrome GWAS Data in Han Chinese. Sci Rep (2015) 5:18855. doi: 10.1038/srep18855 26686423 PMC4685393

[47] Liu Z Li F Pan A Xue H Jiang S Zhu C. CCL19Elevated/Expression During the Disease Process of Primary Sjögren’s Syndrome. Front Immunol (2019) 10:795. doi: 10.3389/fimmu.2019.00795 31068931 PMC6491632

[48] Adnan E Matsumoto T Ishizaki J Onishi S Suemori K Yasukawa M. Human Tolerogenic Dendritic Cells Generated With Protein Kinase C Inhibitor Are Optimal for Functional Regulatory T Cell Induction - A Comparative Study. Clin Immunol (Orlando Fla) (2016) 173:96–108. doi: 10.1016/j.clim.2016.09.007 27658741

[49] Aota K Yamanoi T Kani K Nakashiro K Ishimaru N Azuma M. Inverse Correlation Between the Number of CXCR3 Macrophages and the Severity of Inflammatory Lesions in Sjögren’s Syndrome Salivary Glands: A Pilot Study. J Oral Pathol Med: Off Publ Int Assoc Oral Pathol Am Acad Oral Pathol (2018) 47(7):710–8. doi: 10.1111/jop.12756 29926992

[50] Hjelmervik T Petersen K Jonassen I Jonsson R Bolstad A. Gene Expression Profiling of Minor Salivary Glands Clearly Distinguishes Primary Sjögren’s Syndrome Patients From Healthy Control Subjects. Arthritis Rheum (2005) 52(5):1534–44. doi: 10.1002/art.21006 15880807

[51] Pennec YL Leroy JP Jouquan J Lelong A Katsikis P Youinou P. Comparison of Labial and Sublingual Salivary Gland Biopsies in the Diagnosis of Sjögren's Syndrome. Ann Rheum Dis (1990) 49(1):37–9. doi: 10.1136/ard.49.1.37 PMC1003961 2310226

[52] Devauchellepensec V Mariette X Joussejoulin S Berthelot JM Perdriger A X P. Treatment of Primary Sjögren Syndrome With Rituximab: A Randomized Trial. Nat Rev Rheumatol (2014) 161(5):377–8. doi: 10.7326/M13-1085

[53] Guellec D Cornec D Jousse-Joulin S Marhadour T Marcorelles P Pers JO. Diagnostic Value of Labial Minor Salivary Gland Biopsy for Primary Sjgren’s Syndrome: A Systematic Review. Autoimmun Rev (2013) 12(3):416–20. doi: 10.1016/j.autrev.2012.08.001 22889617

[54] Zhou H Yang J Tian J Wang S. CD8 T Lymphocytes: Crucial Players in Sjögren’s Syndrome. Front Immunol (2020) 11:602823. doi: 10.3389/fimmu.2020.602823 33584670 PMC7876316

[55] Peck A Nguyen C Ambrus J. Early Covert Appearance of Marginal Zone B Cells in Salivary Glands of Sjögren’s Syndrome-Susceptible Mice: Initiators of Subsequent Overt Clinical Disease. Int J Mol Sci (2021) 22(4):1919. doi: 10.3390/ijms22041919 33671965 PMC7919007

[56] Loureiro-Amigo J Palacio-García C Martínez-Gallo M Martínez-Valle F Ramentol-Sintas M Solans-Laqué R. Utility of Lymphocyte Phenotype Profile to Differentiate Primary Sjögren Syndrome From Sicca Syndrome. Rheumatol (Oxf Engl) (2021). doi: 10.1093/rheumatology/keab170 33620072

[57] Matsumura-Kawashima M Ogata K Moriyama M Murakami Y Kawado T Nakamura S. Secreted Factors From Dental Pulp Stem Cells Improve Sjögren’s Syndrome *via* Regulatory T Cell-Mediated Immunosuppression. Stem Cell Res Ther (2021) 12(1):182. doi: 10.1186/s13287-021-02236-6 33726818 PMC7962357

[58] Tedder T Boyd A Freedman A Nadler L Schlossman S. The B Cell Surface Molecule B1 Is Functionally Linked With B Cell Activation and Differentiation. J Immunol (Baltimore Md: 1950) (1985) 135(2):973–9. 3925015

[59] de Rie M Schumacher T van Schijndel G van Lier R Miedema F. Regulatory Role of CD19 Molecules in B-Cell Activation and Differentiation. Cell Immunol (1989) 118(2):368–81. doi: 10.1016/0008-8749(89)90385-7 2463100

[60] Mudd T Lu C Klement J Liu K. MS4A1 Expression and Function in T Cells in the Colorectal Cancer Tumor Microenvironment. Cell Immunol (2021) 360:104260. doi: 10.1016/j.cellimm.2020.104260 33352466 PMC7855947

[61] Tian Q Zhao H Ling H Sun L Xiao C Yin G. Poly(ADP-Ribose) Polymerase Enhances Infiltration of Mononuclear Cells in Primary Sjögren’s Syndrome Through Interferon-Induced Protein With Tetratricopeptide Repeats 1-Mediated Up-Regulation of CXCL10. Arthritis Rheumatol (Hoboken NJ) (2020) 72(6):1003–12. doi: 10.1002/art.41195 31876388

[62] Wang-Renault S Boudaoud S Nocturne G Roche E Sigrist N Daviaud C. Deregulation of microRNA Expression in Purified T and B Lymphocytes From Patients With Primary Sjögren’s Syndrome. Ann Rheumatic Dis (2018) 77(1):133–40. doi: 10.1136/annrheumdis-2017-211417 PMC5754740 28916716

[63] Sellam J Rouanet S Hendel-Chavez H Miceli-Richard C Combe B Sibilia J. CCL19, A B Cell Chemokine, Is Related to the Decrease of Blood Memory B Cells and Predicts the Clinical Response to Rituximab in Patients With Rheumatoid Rrthritis. Arthritis Rheum (2013) 65(9):2253–61. doi: 10.1002/art.38023 23740460

[64] Carubbi F Alunno A Cipriani P Di Benedetto P Ruscitti P Berardicurti O. Is Minor Salivary Gland Biopsy More Than a Diagnostic Tool in Primary Sjögren׳s Syndrome? Association Between Clinical, Histopathological, and Molecular Features: A Retrospective Study. Semin Arthritis Rheum (2014) 44(3):314–24. doi: 10.1016/j.semarthrit.2014.05.015 24935529

[65] Feldman A Law M Grogg K Thorland E Fink S Kurtin P. Incidence of TCR and TCL1 Gene Translocations and Isochromosome 7q in Peripheral T-Cell Lymphomas Using Fluorescence *in Situ* Hybridization. Am J Clin Pathol (2008) 130(2):178–85. doi: 10.1309/pnxuka1cfjmvgcn1 PMC3625137 18628085

[66] Alain F Geneviève DSB Franoise LD. CD3 Deficiencies. Curr Opin Allergy Clin Immunol (2005) 5(6):491–5. doi: 10.1097/01.all.0000191886.12645.79 16264327

[67] Wang Q Li P Wu W. A Systematic Analysis of Immune Genes and Overall Survival in Cancer Patients. BMC Cancer (2019) 19(1):1225. doi: 10.1186/s12885-019-6414-6 31842801 PMC6915928

