# Corrigendum: Integrated Bioinformatics and Validation Reveal Potential Biomarkers Associated With Progression of Primary Sjögren’s Syndrome

**DOI:** 10.3389/fimmu.2021.759203

**Published:** 2021-09-29

**Authors:** Ning Li, Lei Li, Mengyao Wu, Yusi Li, Jie Yang, Yicheng Wu, Haimin Xu, Danyang Luo, Yiming Gao, Xiaochun Fei, Liting Jiang

**Affiliations:** ^1^ Department of Stomatology, Ruijin Hospital, Shanghai Jiao Tong University School of Medicine, College of Stomatology, Shanghai Jiao Tong University, Shanghai, China; ^2^ Department of Pathology, Ruijin Hospital, Shanghai Jiao Tong University School of Medicine, Shanghai, China; ^3^ Core Facility of Basic Medical Sciences, Shanghai Jiao Tong University School of Medicine, Shanghai, China

**Keywords:** primary Sjogren’s syndrome (pSS), transcriptome sequencing, potential biomarkers, bioinformatics analysis, severity

In the original article, there was a mistake in the legends for **Figures 7**, **Figure 8** as published. The authors confused the concept of scanning electron micrographs (SEM) and Transmission electron micrographs (TEM). The correct legend appears below.

**Figure 7**: Representative transmission electron micrograph (TEM) images of acinar-specific ultrastructure changes in the labial salivary gland of non-pSS and pSS. In non-pSS (Grade II), the structure of the acinar is normal, and the glandular epithelial cells in the acinar are myoepithelial cells. The cytoplasmic skeletal components and their unique myofilaments could be seen. With the aggravation of pSS, there is serous and mucous acini atrophy, glandular epithelial cell nucleus shrinkage, organelle swelling to varying degrees, and increase of interstitial collagen components. MD, Mucous Droplets; N, Nucleus; MC, myoepithelial cell; C, collagen; LD, lipid droplets. Bar: 5 μm, 1 μm, 500 nm.

**Figure 8**: Representative transmission electron micrograph (TEM) images of dust-specific ultrastructure changes in the labial salivary gland of non-pSS and pSS. In non-pSS (Grade II), the duct structure of normal glands is clear, and ductal epithelial cells have full nuclei and are connected with myoepithelial cells by desmosomes. As the degree of SS disease increases, duct structures begin to atrophy and become disordered, and part of the basement membrane damage disappears. Ductal epithelial cells can see obvious nuclear pyknosis, fat infiltration in the cytoplasm, and swelling of intracytoplasmic organelles. MC, myoepithelial cell; DN, Ductal Epithelial Cells Nucleus; De, desmosome. Bar, 5 μm, 1 μm, 500 nm.**

In the original article, there was an error. **The authors confused the concept of scanning electron micrographs (SEM) and Transmission electron micrographs (TEM)**.

Corrections of “scanning electron micrographs (SEM)” to “transmission electron micrographs (TEM)” have been made in the following parts: **Abstract, Methods; Introduction, paragraph 4; Materials and Methods, Clinical Samples; and Discussion, paragraph 3**. In addition, **Materials and Methods, Scanning Electron Micrographs (SEM)** has been changed to **Transmission electron micrographs (TEM)**:

“All non-SS and pSS labial salivary glands samples for transmission electron microscopy were subjected to 2.5% glutaraldehyde fixation, 1% osmium tetroxide postfixation, and ethanol gradient dehydration. Two changes of 100% propylene oxido (P.O.) for 10 minutes each and finally into the embedding resin. The samples were sectioned by diamond knife (LEICA EM UC7) to 70-90nm, followed by electron stained with lead citrate, and visualized on a Transmission electron microscope (HITACHI H-7650).”

The authors apologize for these errors and state that they do not change the scientific conclusions of the article in any way. The original article has been updated.

## Publisher’s Note

All claims expressed in this article are solely those of the authors and do not necessarily represent those of their affiliated organizations, or those of the publisher, the editors and the reviewers. Any product that may be evaluated in this article, or claim that may be made by its manufacturer, is not guaranteed or endorsed by the publisher.

