# Supplementary material for: Integrated Bioinformatics and Validation Reveal Potential Biomarkers Associated With Progression of Primary Sjögren’s Syndrome
Source: Front Immunol. 2021 Jul 23;12:697157. doi: 10.3389/fimmu.2021.697157 (PMC8343000; doi:10.3389/fimmu.2021.697157)
Supplement: Supplementary file 1 [file Table_1.docx]

Table S1 Summary of the forward and reverse primers of hub genes used in real-time PCR

| **Gene** | **Forward primers** | **Reverse primers** |
| --- | --- | --- |
| *MS4A1* | CCCATCTACCCAATACTGTTAC | TTCTTTTCCATTCATTCTCAACG |
| *CCL19* | TACTGGCTACCCTTCTCTC | GGATGGGTTTCTGGGTCAC |
| *CD19* | TCTTCTTCCTCCTCTTCCTCAC | GCAGCACAGCGTTATCTCC |
| *TCL1A* | GAGATAAAGGATAGGTTACAGTTAC | ATCGGTATCGTCCATCAGG |
| *CXCL9* | ATCTTGCTGGTTCTGATTGGAG | CCTTGGTTGGTGCTGATGC |
| *CD3D* | TACTGGCTACCCTTCTCTC | CACCGTTCCCTCTACCC |
| *CD3G* | CCTGGCTGTCCTCATCCTG | CTTCTTGATAGTCATACACCTTAACC |
| *actin* | CATGTACGTTGCTATCCAGGC | CTCCTTAATGTCACGCACGAT |

Table S2 KEGG analyses of DEGs(selected top 11 according to adjusted P value)

| ID | Description | p.adjust | Gene ID | Count |
| --- | --- | --- | --- | --- |
| hsa04061 | Viral protein interaction with cytokine and cytokine receptor | 2.10E-13 | CXCL13/CCL19/CXCL9/CXCR4/TNF/CCR7/CCL18/CCR6/CCL5/CCR1/CCR5/CXCL10/CXCL11/IL2RB/XCL1/CCL13/XCL2 | 17 |
| hsa04062 | Chemokine signaling pathway | 1.79E-11 | CXCL13/CCL19/CXCL9/CXCR4/ADCY7/ITK/STAT1/CCR7/CCL18/CCR6/CCL5/PLCG2/CCR1/CCR5/CXCL10/RAC2/CXCL11/XCL1/CCL13/XCL2 | 20 |
| hsa04060 | Cytokine-cytokine receptor interaction | 1.13E-09 | CXCL13/CCL19/TNFRSF17/CXCL9/CXCR4/TNF/IL21R/CCR7/CCL18/CCR6/CCL5/LTB/CCR1/CCR5/CXCL10/CXCL11/IL2RB/BMP6/XCL1/CCL13/CD27/XCL2 | 22 |
| hsa05169 | Epstein-Barr virus infection | 1.77E-08 | HLA-DRA/B2M/CD247/CD3D/ISG15/CD19/CD3G/BTK/TNF /STAT1/OAS2/TAP1/OAS3/PLCG2/CXCL10/HLA-C/HLA-F | 17 |
| hsa04064 | NF-kappa B signaling pathway | 8.07E-08 | CCL19/BTK/TNF/BCL2A1/LTB/PLCG2/TLR4/VCAM1/BIRC3/LCK/LY96/CCL13 | 12 |
| hsa04660 | T cell receptor signaling pathway | 7.03E-07 | CD247/CD3D/CD3G/TNF/ITK/CD28/LCP2/ICOS/CD8A/LCK/RASGRP1 | 11 |
| hsa05340 | Primary immunodeficiency | 1.87E-06 | CD3D/CD19/BTK/ICOS/TAP1/CD8A/LCK | 7 |
| hsa04514 | Cell adhesion molecules | 4.04E-06 | HLA-DRA/CD2/ICAM2/CD28/ICOS/ITGA4/PECAM1/VCAM1/ CD8A/HLA-C/TIGIT/HLA-F | 12 |
| hsa04621 | NOD-like receptor signaling pathway | 5.62E-06 | AIM2/IFI16/TNF/STAT1/GBP1/OAS2/CYBB/CCL5/GBP5/OAS3/TLR4/BIRC3/CARD16 | 13 |
| hsa04650 | Natural killer cell mediated cytotoxicity | 6.94E-06 | CD247/SH2D1A/TNF/ICAM2/LCP2/PLCG2/MICB/CD48/HLA-C/RAC2/LCK | 11 |
| hsa05235 | PD-L1 expression and PD-1 checkpoint pathway in cancer | 0.000174 | CD247/CD3D/CD3G/STAT1/CD28/TLR4/LCK/RASGRP1/BATF | 9 |

Table S3 GO-BP analyses of up-regulated DEGs(selected top 11 according to adjusted P value)

| ID | Description | p.adjust | Gene ID | Count |
| --- | --- | --- | --- | --- |
| GO:0009615 | response to virus | 1.10E-20 | CCL19/IFI27/MX1/IFITM1/ISG15/IFI6/NLRC5/IFI44L/CXCL9/ISG20/RTP4/AIM2/CXCR4/IFI16/TNF/STAT1/SAMHD1/GBP1/OAS2/PMAIP1/HERC5/CCL5/PARP9/IFIT3/FGL2/TRIM22/IFIT2/OAS3/IFIT5/IFIT1/IRF5/MICB/APOBEC3G/IFI44/BIRC3/CXCL10/IFITM2/XCL1/ | 38 |
| GO:0050851 | antigen receptor-mediated signaling pathway | 1.43E-16 | LAX1/HLA-DRA/CD247/TRAT1/CD3D/MS4A1/CD19/CD3G/PTPN22/CD38/PLCL2/BTK/KLHL6/ITK/CCR7/CD28/GBP1/ LCP2/MEF2C/MNDA/PLCG2/GCSAM/IGHV1-69/PAX5/FCRL3/ STAP1 / PVRIG /IGHM/ LCK/ UBASH3A/IGLC1/BLK/BTN1A1 | 33 |
| GO:0002429 | immune response-activating cell surface receptor signaling pathway | 9.82E-15 | LAX1/HLA-DRA/CD247/TRAT1/CD3D/MS4A1/CD19/CD3G/ PTPN22/CD38/PLCL2/BTK/KLHL6/ITK/ICAM2/CCR7/CD28/GBP1/LCP2/MEF2C/MNDA/PLCG2/MICB/GPLD1/GCSAM/IGHV1-69/IGLV6-57/PAX5/FCRL3/STAP1/PVRIG/IGHM/LCK/UBASH3A/ IGLC1/BLK/BTN1A1 | 37 |
| GO:0060337 | type I interferon signaling pathway | 2.47E-14 | IFI27/MX1/IFITM1/ISG15/IFI6/NLRC5/ISG20/XAF1/STAT1/SAMHD1/OAS2/IFIT3/IFIT2/OAS3/IFIT1/IRF5/HLA-C/IFITM2/HLA-F | 19 |
| GO:0030098 | lymphocyte differentiation | 8.39E-14 | CCL19/RHOH/B2M/CD2/CD3D/MS4A1/CD19/CD3G/PTPN22/PLCL2/DOCK11/BTK/ITK/EOMES/CCR7/CCR6/CD28/MYB/FGL2/ITGA4/PLCG2/DOCK10/VCAM1/CD8A/FCRL3/LAG3/LEF1/LCK/RASGRP1/CD27/BATF | 31 |
| GO:0042110 | T cell activation | 9.01E-14 | LAX1/CCL19/RHOH/B2M/CD2/SIT1/CD3D/CD3G/PTPN22/ITK/EOMES/CCR7/CCR6/CD28/CCL5/ICOS/MYB/FGL2/BTLA/AIF1/MICB/VCAM1/APBB1IP/CD8A/RASAL3/RAC2/LAG3/LEF1/LCK/TIGIT/RASGRP1/CLECL1/XCL1/CD27/BATF | 35 |
| GO:0050852 | T cell receptor signaling pathway | 1.99E-07 | HLA- DRA/CD247/TRAT1/CD3D/CD3G/PTPN22/BTK/ITK/CCR7/CD28/GBP1/LCP2/PLCG2 /PVRIG/LCK/UBASH3A/BTN1A1 | 17 |
| GO:0042113 | B cell activation | 6.95E-12 | BANK1/LAX1/MS4A1/CD19/CD38/SAMSN1/PLCL2/DOCK11/BTK/CCR6/CD28/MEF2C/MNDA/ITGA4/PLCG2/DOCK10/TLR4/VCAM1/IGHV1-69/FCRL1/FCRL3/LEF1/IGHM/GAPT/IGLC1/CD27/BATF | 27 |
| GO:0050853 | B cell receptor signaling pathway | 3.13E-13 | MS4A1/CD19/PTPN22/CD38/PLCL2/BTK/KLHL6/ITK/MEF2C/MNDA/PLCG2/GCSAM/IGHV1-69/PAX5/FCRL3/STAP1/IGHM/LCK/IGLC1/BLK | 20 |
| GO:0022407 | regulation of cell-cell adhesion | 3.42E-08 | CXCL13/LAX1/CCL19/PTPN22/TNF/CCR7/CD28/CCL5/ICOS/MYB/FGL2/BTLA/AIF1/ITGA4/VCAM1/RASAL3/LAG3/LEF1/LCK/TIGIT/RASGRP1/CLECL1/BMP6/XCL1/CD27 | 25 |
| GO:1990868 | response to chemokine | 8.39E-10 | CXCL13/CCL19/CXCL9/CXCR4/CCR7/CCL18/CCR6/CCL5/CCR1/CCR5/CXCL10/CXCL11/XCL1/CCL13/XCL2 | 15 |

Table S4 GO-MF analyses of up-regulated DEGs(selected top 9 according to adjusted P value)

| ID | Description | p.adjust | Gene ID | Count |
| --- | --- | --- | --- | --- |
| GO:0042379 | chemokine receptor binding | 5.00E-07 | CXCL13/CCL19/CXCL9/STAT1/CCL18/CCL5/CXCL10/CXCL11/XCL1/CCL13/XCL2 | 11 |
| GO:0015026 | coreceptor activity | 0.000234 | CXCR4/CD28/ITGA4/CCR5/CD8A/FCRL1/LY96 | 7 |
| GO:0003823 | antigen binding | 0.000251 | HLA-DRA/SLAMF1/TAP1/ITGA4/CD48/IGHV1-69/IGLV6-57/HLA-C/LAG3/IGHM/HLA-F/IGLC1 | 12 |
| GO:0045236 | CXCR chemokine receptor binding | 0.000637 | CXCL13/CXCL9/CXCL10/CXCL11 | 4 |
| GO:0005126 | cytokine receptor binding | 0.000676 | CXCL13/CCL19/CXCL9/TNF/STAT1/CCL18/CCL5/LTB/CXCL10/CXCL11/STAP1/BMP6/XCL1/CCL13/XCL2 | 15 |
| GO:0003950 | NAD+ ADP-ribosyltransferase activity | 0.004742 | PARP8/PARP9/TIPARP/PARP14 | 4 |
| GO:0016298 | lipase activity | 0.028156 | PLCL2/PLA2G7/CCL5/PLCG2/CCR1/CCR5/GPLD1 | 7 |
| GO:0023023 | MHC protein complex binding | 0.044093 | HLA-DRA/MS4A1/CD8A | 3 |
| GO:0005070 | SH3/SH2 adaptor activity | 0.049714 | SH2D1A/CD28/FCRL2/STAP1 | 4 |

Table S5 GO-CC analyses of up-regulated DEGs(selected top 8 according to adjusted P value)

| ID | Description | p.adjust | Gene ID | Count | |
| --- | --- | --- | --- | --- | --- |
| GO:0009897 | external side of plasma membrane | 1.65E-13 | B2M/CD2/CD3D/MS4A1/CXCL9/CD19/CD3G/CXCR4/TNF/CCR7/CCR6/CD28/ICOS/CD163/PECAM1/CCR1/MICB/TLR4/VCAM1/CCR5/CD69/CD8A/IGHV1-69/CXCL10/HLA-C/LAG3/IGHM/HLA-F/IGLC1/IL2RB/CD27/BTN1A1 | | 32 |
| GO:0030670 | phagocytic vesicle membrane | 0.011648 | B2M/CYBB/RAB8B/TAP1/HLA-C/RAC2/HLA-F | | 7 |
| GO:0001772 | immunological synapse | 0.011694 | RHOH/GZMA/CD53/CD28/LCK | | 5 |
| GO:0042611 | MHC protein complex | 0.024125 | HLA-DRA/B2M/HLA-C/HLA-F | | 4 |
| GO:0001891 | phagocytic cup | 0.024125 | TNF/BIN2/AIF1/TLR4 | | 4 |
| GO:0030666 | endocytic vesicle membrane | 0.024125 | HLA-DRA/B2M/CYBB/RAB8B/TAP1/CD163/HLA-C/RAC2/HLA-F | | 9 |
| GO:0000779 | condensed chromosome, centromeric region | 0.049276 | NUP37/MAD2L1/NDC80/CENPE/BUB1/RASSF2/NCAPG | | 7 |
| GO:0000775 | chromosome, centromeric region | 0.050413 | NUP37/MAD2L1/NDC80/OIP5/CENPE/BUB1/RASSF2/NCAPG/DYNLL1 | | 9 |
